# The role of mutational analysis of KIT and PDGFRA in gastrointestinal stromal tumors in a clinical setting

**DOI:** 10.1186/1479-5876-9-75

**Published:** 2011-05-23

**Authors:** Alessandra Maleddu, Maria A Pantaleo, Margherita Nannini, Guido Biasco

**Affiliations:** 1 "L.&A.Seragnoli" Department of Hematology and Oncological Sciences, S.Orsola-Malpighi Hospital, University of Bologna, Italy; 2 "G. Prodi" Interdepartmental Centre of Cancer Research, University of Bologna, Italy

## Abstract

Gastrointestinal stromal tumors (GIST) are the most common mesenchymal tumors of the gastrointestinal tract. Most GIST harbor a mutation affecting either the KIT or PDGFRA genes, whereas a small subgroup of tumors is wild type for mutations.

Mutation of tyrosine kinase receptors is a mechanism of drug resistance that can occur either at the beginning of treatment (primary resistance) or during the course of therapy (secondary resistance). In addition, mutational status can predict the response to treatment with tyrosine kinase inhibitors, but the role of mutational status as a prognostic factor remains controversial.

Evidence of a potential role of mutational status as a prognostic factor has emerged over the past decade. The presence of KIT exon 11 insertion/deletion involving either one or both Trp557-Lys558 amino acids correlates with a poorer clinical outcome if compared to patients with tumors wild type for KIT exon 11 mutations. A malignant clinical behavior has also been documented for KIT exon 13 and KIT exon 9 mutant GIST. Patients with GIST harboring a PDGFRA mutation seem to have a better prognosis than the others.

The aim of this paper is to review the clinical significance of tyrosine kinase mutational status.

## Introduction

Gastrointestinal stromal tumors (GIST) are rare tumors of the gastrointestinal tract. They arise mostly in the stomach, followed by the small bowel and colon. Less frequently they are found in the rectum, esophagus or in an extra-gastrointestinal location. The biology of GIST has been widely investigated since Hirota et al. [1] demonstrated mutations of the KIT receptor as a pathogenic mechanism of GIST. Other mutations affecting KIT exons 9, 13 and 17 have been demonstrated [2,3]. About 15% of GIST do not express KIT mutations and of these approximately 5 to 7% have a mutation affecting the gene encoding for PDGFRA [4]. There is also a small subgroup of GIST, called wild type (WT), which do not harbor either KIT or PDGFRA mutations [5].

KIT and PDGFRA are two trans-membrane receptors that belong to the type III tyrosine kinase family whose natural ligands are stem cell factor (SCF) and platelet-derived growth factor (PDGF). Both receptors have a similar structure with five immunoglobulin-like domains located on the extracellular side of the receptor, a trans-membrane portion and an intracellular part containing two tyrosine kinase domains: one with an adenosine triphosphate (ATP) binding region and the other with a phosphotransferase region (activation loop). Activation of the receptor normally occurs with ligand binding which triggers the receptor dimerization, the autophosphorylation of the tyrosine kinase domain and finally the activation of substrates like PI3K/Akt, Ras/MAPK and JAK/STAT. This promotes cell cycle activation, cell proliferation, and apoptosis inhibition [6,7]. Several gain-of-function mutations of KIT and PDGFRA affecting different exons have been reported [8,9].

The correlation between KIT and PDGFRA mutational status and the response to tyrosine kinase inhibitors and their role in primary and secondary resistance has been widely investigated [10,11].

The aim of this paper is to review the clinical significance of mutational status and its value as a predictive/prognostic factor in limited and metastatic disease.

## Prognostic value of mutational analysis in localized GIST

Whenever possible surgery is the best treatment for GIST. Unfortunately, even after radical surgery the five-year survival rate is about 54% and the disease-free survival (DFS) is 45% [12,13]. Tumor size (≥10 cm), mitotic rate (≥5/50HPF) and tumor location are known to be independent prognostic factors for shorter DFS in fully resected GIST patients. In 2002 Fletcher et al. developed a risk stratification for primary tumors (National Health Institute -NHI classification), considering tumor size and mitotic count as predictive factors of aggressive behavior [6]. In 2006 Miettinen and Lasota analyzed the follow-up data from more than 1600 fully resected tumors and, on the basis of their results, revised the NIH classification adding primary tumor location as an important prognostic factor to identify the class of risk for resected primary GIST [7]. According to the latest classification, the risk of recurrence goes from being very low for small tumors (≤ 2 cm) with low mitotic rate (≤5/50HPF) and gastric location, to close to 90% for large tumors (> 10 cm) with high mitotic rate (≥ 5/50HPF) and small intestinal location [7]. Due to a wide spectrum of behavior, it is crucial to find further factors that can have a prognostic value in predicting the risk of relapse for fully resected tumors. The importance of the mutational status of KIT and PDGFRA as a prognostic factor remains controversial, although its predictive value on tyrosine kinase inhibitors response is now clearer.

Early evidence of a potential role of mutational status as a prognostic factor appeared in the late nineties, when different groups observed a correlation between KIT exon 11 mutations and a poorer clinical outcome compared to patients with tumors WT for KIT exon 11 mutations. Ernst et al. identified a subgroup of 13 KIT exon 11 mutant tumors in a larger group of 35 GIST patients and observed that the mutation was associated with a shorter survival rate (p = 0.001). No correlation between mutations, tumor size or mitotic index was observed [14]. When GIST were still classified as malignant or benign, KIT exon 11 mutations were noticed to be more common in the malignant subtype [15]. In addition, a study of 124 GIST patients showed a clear difference in prognosis for patients with or without KIT exon 11 mutations and a subdivision into mutation-positive and mutation-negative patients was proposed [16]. However, in 1999 only KIT exon 11 was studied and the prognostic role of mutations could not be evaluated.

Malignant clinical behavior was also documented for KIT exon 9 and KIT exon 13 mutant GIST [16-18], and for the first time the association between KIT exon 9 mutation and small intestinal location was reported [17]. In general, all the cited studies focused on KIT exon 11 mutations, whereas few dealt with KIT exon 9, 13 or 17 mutations. The first study to screen KIT exons 9, 11, 13, and 17 for mutations was performed in 2002 by Singer et al.: 44 tumors out of 48 harbored a KIT mutation. They found a KIT mutation was associated with a poor clinical outcome and they also hypothesized that specific KIT mutations could have a prognostic value. The multivariate analysis showed that patients with GIST harboring a KIT deletion/insertion had a significantly shorter recurrence-free survival (RFS) than patients with tumors harboring a KIT exon 11 missense mutation, which was more common in favorable-outcome, low-grade GIST [19]. A larger study on 120 patients identified two small subgroups with different clinical outcomes [20]. The first small subgroup of eight cases had a more favorable prognosis and consisted of tumors harboring an insertion of 6-20 amino acids representing intra-tandem-duplications (ITDs) at the 3' end of exon 11. All eight tumors were located in the stomach, had a spindle cell morphology, and a low mitotic count. Patients were all older than 60 years of age and seven out of eight were female. The second subgroup included 13 tumors that harbored a KIT exon 9 mutation, had a predominant small intestinal location and a poorer outcome compared to the other patients [18].

In 2005 a large retrospective study by Kim et al. enrolled 86 patients who underwent radical resection of localized GIST [21]. Sixty-one GIST had a mutation of KIT exon 11 and three had a mutation of KIT exon 9. KIT exons 13 and 17 were screened but no mutations were found in 22 tumors. The class of risk considering tumor size and the mitotic rate was identified for all the patients. All three KIT exon 9 mutations were insertions of six nucleotides, resulting in duplication of Ala502-Tyr503, two patients had a high risk GIST but all three KIT exon 9 mutants had a relapse of the disease. The most common mutation of KIT exon 11 was a deletion that involved codons between 550 and 570. Three IDTs were also found in tumors with spindle cell morphology, no mitotic activity and benign clinical behaviors. The patients were all female and were all alive and relapse-free after 24-80 months of follow-up, despite the tumor risk class. In general the five-year RFS for patients whose tumor harbored a KIT mutation was significantly shorter than for patients with tumors without KIT mutations. KIT mutations were also observed to be associated with a higher mitotic rate, which together with tumor size was already a known negative prognostic factor [21].

Wardelmann et al. correlated the mutational status of 55 GIST and the clinical outcome. The size of 50 tumors was known, 21 tumors had a diameter ≤ 5 cm and none of them had evidence of metastasis, 29 tumors had a diameter larger than 5 cm and 15 of them had a metastatic spread. A mutation affecting 557 and/or 558 codons of KIT exon 11 was found in 13 of 15 metastatic tumors and in only two of the nine mutated and not metastatic tumors. These results suggested that mutations involving 557 and/or 558 codons could be used as an additional parameter to estimate poor survival [22]. Subsequent studies confirmed the hypothesis of an association between a KIT exon 11 insertion/deletion involving either one or both Trp557-Lys558 amino acids and a poor clinical outcome [23,24]. DeMatteo et al. studied a series of 127 non-metastatic GIST patients who underwent complete tumor resection and were all entered in a follow-up program [24]. As expected, tumor size, mitotic rate and location predicted the RFS in the multivariate analysis. Only in the univariate analysis did KIT exon 9 mutations and KIT exon 11 deletions involving codons 557 and/or 558 correlate with a higher rate of recurrence, whereas patients with point of mutation or insertion of KIT exon 11 had a lower rate of recurrence, and patients with WT tumors had an intermediate outcome. Only four patients had a GIST harboring a mutation of KIT exon 9, they all had a disease recurrence but the number was too small to hypothesize a prognostic value [24].

The Spanish Group for Sarcoma Research (GEIS) selected 162 patients who underwent complete resection of localized GIST between 1994 and 2001. All the tumors were ≥ 2 cm and KIT positive at immunohistochemical analysis. They evaluated the prognostic value of RFS prediction of different KIT and PDGFRA mutations. The results were analyzed when the median follow-up was 42 months and at that point 41 of the 162 patients experienced a disease recurrence and the five-year RFS was 68%. According to the NIH and the Miettinen-Lasota risk classifications, tumors with a high mitotic count and large dimensions had a significantly shorter RFS. In addition, following the Miettinen-Lasota revised risk classification the RFS was significantly shorter for patients whose primary tumor was located in the small bowel compared to patients whose primary tumor was located in the stomach. A very interesting statistic was the higher recurrence rate (5-year RFS 57% ± 13%) of tumors harboring mutations of the KIT gene, rather than tumors without KIT mutations (5-year RFS 80% ± 11%). In the univariate analysis, patients with deletions involving codons 557 and/or 558 of KIT exon 11 had a less favorable outcome than patients with different mutations or without KIT mutations. The presence of deletions involving codons 557 and/or 558 of KIT exon 11 was also significantly associated with a higher rate of recurrence in the multivariate analysis together with size, mitotic count and high cellularity, which are known prognostic factors [25].

After a longer follow-up, the data from the same group of 162 patients were analyzed again by the Spanish Group for Sarcoma Research (GEIS). The first analysis demonstrated that the mutations within KIT exon 11 involving codons 557 and/or 558 have a prognostic relevance. The objective of the new study was to demonstrate if critical deletions still are an independent prognostic factor after a longer follow-up, and if there were any time-related prognostic factors for RFS. When the analysis was performed the median follow-up was 84 months, the factors assessed were the class of risk (both classifications NIH and Miettinen-Lasota were considered) and the type of mutation. Mutations were also classified as deletions of codons 557 and/or 558 of KIT exon 11 (critical mutations), non-deletion-type mutations of KIT exon 11 (NDTM) which included missense mutations and insertions, and other deletions of KIT exon 11. Results showed that, for the first four years after surgery and for the entire seven-year follow-up the presence of critical deletions belonging to a high-risk category were independent prognostic factors for RFS. In the first 4 years after surgery only the high-risk category of the Miettinen-Lasota classification and NDTM were independent prognostic factors for RFS. In fact, the presence of critical mutations could be useful to identify a subset of patients with a higher risk of relapse in the first four years after surgery, whereas the presence of NDTM could identify a subset of patients more likely to experience a relapse beyond three or four years after surgery [26].

A better outcome and a lower chance of metastasis seem to be associated with PDGFRA exon 18 mutations [27]. Lasota et al. screened 1000 GIST for KIT exon 11 mutations, KIT exon 9 (only the non-gastric tumors) and PDGFRA exons 18 and 12. PDGFRA mutant tumors had a prevalent gastric location, epithelioid morphology (pure or prevalent) and low mitotic count. Of 1000 GIST, a PDGFRA exon 18 mutation was found in 122 of the 346 gastric tumors and only two of the 75 small intestinal tumors. Ten of the 170 gastric tumors and one of the 54 small intestinal tumors had a PDGFRA exon 12 mutation. One hundred and five of those had ≤5 mitosis/50HPF, and 40 had no mitotic activity. Clinical data were available for 91 out of 128 PDGFRA exon 18 mutant tumors. After a median follow-up of 135 months, 41 were alive and with no evidence of disease, 24 had died from other causes, 16 had died for unknown causes, and ten had a progressive disease. Seven died of the disease and three were still alive at the end of the follow-up (370 months). The authors concluded that 83% of GIST with PDGFRA mutations have a good prognosis [27]. The same group of scientists demonstrated later that PDGFRA exon 14 mutant GIST are mostly gastric, have epithelioid morphology and benign clinical behavior [28].

## Predictive value of response to therapy

Although the role of KIT/PDGFRA mutational status as a prognostic factor is controversial, it is well known to predict the response to treatment with tyrosine kinase inhibitors.

The mutation of tyrosine kinase receptors is a mechanism of drug resistance occurring either at the beginning of treatment (primary resistance) or during the course of therapy (secondary resistance).

The first study that showed the correlation between the response to imatinib at a dose of 400 mg/day and mutational status in GIST was performed by Heinrich et al. One hundred and twenty-seven patients with metastatic GIST received imatinib, 71 had a tumor with a mutation of KIT exon 11, 23 of KIT exon 9, two of KIT exon 13 and KIT exon 17; PDGFRA exon 18 was mutated in six cases and nine tumors were WT. The clinical response varied considering the different mutations, the stronger predictor of response being any KIT exon 11 mutation. The 87.5% of patients whose tumor had a KIT exon 11 mutation achieved a partial response, whereas only 47.8% of patients whose tumor had a KIT exon 9 mutation had a partial response [10].

The European Organization for Research and Treatment of Cancer (EORTC) phase I and II studies [29-31] enrolled patients with metastatic GIST and tested the safety of imatinib given at a dose of 400 mg/day or 800 mg/day and investigated its activity. The results of the mutational analysis performed on 37 tumor specimens showed a further correlation between certain mutations and their response to imatinib. Of the 37 tumors, 24 had a KIT exon 11 mutation, four had a KIT exon 9 mutation, one had a KIT exon 13 mutation and two had a PDGFRA exon 18 mutation. Patients whose tumors had a KIT exon 11 mutation had a higher partial response rate than the others and patients whose tumors harbored a KIT mutation enjoyed a longer median survival time and a lower recurrence rate [32].

Two recent randomized phase III studies compared the outcome of metastatic GIST patients treated with imatinib 400 mg/day or 800 mg/day. The EU-AUS trial (EORTC and Australian Gastro-Intestinal Trial Group) enrolled 946 patients with metastatic GIST between 2001 and 2002. The primary endpoint of the study was RFS and the patients were randomized to receive imatinib at the two doses with the possibility of cross over to the higher doses in case of progressive disease [33]. Mutational analysis was performed in 377 cases: 248 harbored a KIT exon 11 mutation, 58 a KIT exon 9 mutation, six a KIT exon 13 mutation, and three a KIT exon 17 mutation, whereas ten tumors harbored a PDGFRA exon 18 mutation. Patients with tumors expressing any mutation of KIT exon 11 had a higher response rate and a longer median survival than patients whose tumors harbored KIT exon 9 mutations or whose tumors were WT. Once the KIT exon 11 tumor mutant group was divided into subgroups, the statistical analysis revealed a poorer outcome for patients whose tumor had large exon 11 deletions, especially if involving codons 577-579, this may be due to the conformational change in the receptor. In the group of KIT exon 9 mutant tumors the response rate was significantly higher for patients enrolled in the 800 mg/day arm [33].

The other phase III study (US-CDN) was conducted by the Southwest Oncology Group (SWOG), Cancer and Leukemia Group B (CALGB), National Cancer Institute of Canada (NCI-C) and Eastern Cooperative Oncology Group (ECOG) [34]. This study, which had OS as primary endpoint, enrolled 746 patients with advanced GIST and randomized them to receive imatinib 400 mg/day or 800 mg/day equal to the EU-AUS trial. Of the total, 428 tumors were screened for KIT and PDGFRA mutations. The analysis disclosed a KIT exon 11 mutation in 283 cases, a KIT exon 9 mutation in 32 cases, whereas 67 tumors were WT. Patients were randomly assigned to receive imatinib at the daily dose of 400 mg or 800 mg. The time to progression (TTP) did not change for patients with any KIT exon 11 mutation or WT GIST. Patients with a KIT exon 9 mutation had a significantly higher rate of response if treated with imatinib at the daily dose of 800 mg, but there was no difference in time to progression and overall survival between the two groups. No differences were observed for KIT exon 11 mutant or WT GIST treated with 400 or 800 mg/day. Instead, any KIT exon 11 mutation was associated with a better outcome in patients with advanced GIST treated with imatinib compared to patients with KIT exon 9 mutations or WT tumors [34].

The Gastrointestinal Stromal Tumor Meta-Analysis Group (MetaGIST) re-analyzed and compared the data from the EU-AUS and the US-CDN studies to confirm the results, validate the suggested prognostic and predictive factors and to explain the differences between results in the two studies by reviewing the characteristics of the two populations.

A small advantage on PFS was observed for the high-dose arm in both studies, no difference in overall survival (OS). Prognostic factors for PFS and OS were then considered. Mutational status was a significant prognostic factor (p < 0.0001) for PFS, and patients with KIT exon 11 mutation had a more favorable prognosis than those with exon 9 mutation or WT.

Exon 9 mutation was the only significant predictive factor for a benefit of high-dose therapy. Patients with KIT exon 9 mutations treated with imatinib 800 mg/day had a significantly longer PFS than the others and the estimated rate of progression or death was also significantly decreased (p = 0.017) [35].

A further phase III trial compared two doses of imatinib for treatment of unresectable advanced GIST. To investigate the influence of mutational status on imatinib response, 128 GIST specimens from those patients were screened for KIT and PDGFRA mutations. The estimated median survival was 63 months for patients whose tumor harbored a KIT exon 11 mutation, 44 months for patients whose tumors harbored a KIT exon 9 mutation and 26 months in case of other mutations or WT GIST. In addition, a mutation within KIT exon 11 was associated with a better outcome for the first 30 months of therapy [36].

Fewer studies have defined the role of mutational status as a prognostic and predictive factor in the adjuvant setting. It is well known that adjuvant therapy with imatinib is associated with a longer RFS [37]. For this purpose 713 patients who underwent complete resection of a primary GIST were enrolled on the Z9001 study. All primary tumors were ≥3 cm and expressed KIT. Patients were randomized to receive imatinib 400 mg/day for one year or a placebo. Tumor size, mitotic index and mutational status were available for 513 patients. After a median follow-up of 20 months, the two-year RFS was 74% in the placebo arm vs. 91% in the imatinib arm. The two-year RFS for patients with KIT exon 11 mutation was 65% vs. 91% (placebo and imatinib arm respectively p < 0.0001), 76% vs. 100% for the PDGFRA mutation (p < 0.01), whereas there were no difference in overall RFS for KIT exon 9 mutation, but the one-year RFS was shorter for patients in the placebo arm (80%) than in the imatinib arm (100%). This study showed that mutational status together with pathological features have a prognostic and predictive value for RFS after complete surgical resection of primary GIST [38].

Secondary resistance occurs after a median period of 24 months of treatment with imatinib. There are several mechanisms involved in resistance like the activation of an alternative downstream signaling pathway such as AKT/mTOR, the activation of an alternate tyrosine kinase receptor and the loss of KIT expression, the genomic amplification of KIT, and the gain of new KIT/PDGFRA mutations [39]. New KIT/PDGFRA mutations are currently considered the most important and the most common mechanism [40-43].

Mutational analysis performed on tissue specimens from resistant lesions disclosed secondary acquired mutations developed during imatinib therapy. The frequency of secondary mutations is over 50% in those tumors with primary KIT exon 9 or 11 mutations. Secondary mutations are single substitutions and occur in different exons but on the same allele of the primary mutation. Similar to chronic myeloid leukemia, acquired imatinib resistant mutations affect the tyrosine kinase domain and the activation loop, encoded by exons 13, 14 and 17 respectively [9,40,43-47]. The most common secondary mutation is the V654A, mainly found in GIST harboring an exon 11 primary mutation [48]. PDGFRA secondary mutations are rare. The D842V was identified in one patient with primary mutation V561D, which is known to be associated with imatinib resistance [48]. In general, secondary mutations were detected only in progressive nodules and not in non-progressive ones [46,49]. In addition, patients with WT GIST do not develop secondary mutations [46]. A recent study by Liegl et al. analyzed 53 metastases from 14 patients after imatinib or sunitinib treatment failure. Primary tumors included GIST with classical features (KIT positive, and mutated on KIT exons 9, 11, or 13), but also KIT negative tumors, GIST with unusual morphology, and KIT/PDGFRA WT GIST. Secondary KIT mutations were found in nine out of 11 GIST with KIT primary mutation. Two to five different mutations were found in different metastases I six out of nine patients, and in three out of nine patients two mutations were found in one or more tumor samples. Five recurrent points of mutation were located in the KIT tyrosine kinase domain and in the ATP activation loop. No secondary mutations were found in KIT/PDGFRA WT GIST or in those with unusual morphology [47].

Sunitinib inhibits double mutant GIST and the response to therapy is influenced by the mutational status [11,50]. Seventy-eight imatinib resistant patients were treated with sunitinib, 58% of patients had tumors harboring KIT exon 9 mutations, 34% had tumors harboring KIT exon 11 mutations, and 56% of tumors were WT. Results showed a significantly longer progression-free survival and overall survival for patients with primary KIT exon 9 mutations (p < 0.0005) or WT (p < 0.0356) than for those with KIT exon 11 mutations. In addition, patients whose tumor expressed a secondary mutation affecting exons 13 or 14 had a better outcome than those whose tumor had a KIT exon 17 or 18 mutation [11].

In addition to the cellular and mutational profile of the disease, broad variations of imatinib plasma levels have been monitored in GIST patients [51,52]. The decrease of imatinib bioavailability during chronic therapies should be considered a further possible mechanism of resistance. Recently Demetri et al. studied the imatinib pharmacokinetic and pharmacodynamic profiles in advanced GIST patients to detect possible correlations between the imatinib plasma concentrations and clinical outcome. They observed that patients with the lowest imatinib serum levels had the lowest overall response rate and the shortest time to progression [51,52].

## Conclusions

The value of mutational status as a predictive and prognostic factor for RFS in metastatic GIST treated with imatinib is clear. What is now becoming more evident is its potential role as a predictive and prognostic factor for resected GIST treated with imatinib. Tumors harboring KIT exon 11 mutations have a better outcome under imatinib treatment at a dose of 400 mg/day than tumors harboring different mutations, and KIT exon 9 mutant tumors have a longer RFS if treated with the high dose of imatinib, corresponding to 800 mg/day. Patients with KIT exon 9 mutant tumors achieve a better response to sunitinib, than those with exon 11 mutations. The PDGFRA exon 18 D842V point mutant activates PDGFRA both in vitro and in vivo [10,50] and is also imatinib resistant in vivo and in vitro [30,53,54], whereas other mutations affecting exon 18 (D846Y, N848K, Y849K and HDSN845-848P) are imatinib sensitive [53].

In the adjuvant setting, available data show that mutational status can be considered a predictive and prognostic factor for GIST patients treated with imatinib after radical surgery [38].

Dei Tos et al. recently reviewed a series of 929 untreated GIST to correlate the natural history of disease with pathological features, but the mutational status of tumors was not available [55]. Tumor size (≥10 cm), mitotic rate (≥5/50HPF) and tumor location are the only recognized independent prognostic factors for GIST patients [11,12], but unfortunately it is still unclear whether or not mutational status could be an independent prognostic factor for disease recurrence in untreated patients. It would be useful to be able to study mutational status on a large population of untreated GIST but this has become more difficult since evidence emerged of a longer RFS following adjuvant treatment with imatinib.

Lastly, knowledge of the predictive and prognostic value of mutational status could lead physicians to establish the dose of imatinib, identify those patients who would not benefit from imatinib treatment (PDGFRA exon 18 D842V mutant tumors are imatinib resistant), chose a second line therapy, and evaluate the different risk of relapse during follow-up. These data emphasize that mutational status must play a predominant role in the clinical management of patients and that new findings are necessary to establish the mechanisms responsible for imatinib resistance in specific subsets of tumors like PDGFRA D842V mutant and WT GIST.

## Abbreviations

WT: Wild Type; ATP: Adenosine Triphosphate; PDGFRA: Platelet Derived Growth Factor Receptor Alpha; TKI: Tyrosine Kinase Inhibitor; DFS: Disease Free Survival; NHI: National Health Institute; HPF: High Power Field; ITDs: Intra-Tandem-Duplications; RFS: Recurrence-Free Survival; NDTM: Non-Deletion-Type-Mutations; OS: Overall Survival; PFS: Progression-Free Survival.

## Competing interests

The authors declare that they have no competing interests.

## Authors' contributions

AM designed the study, carried out the acquisition and participated in data interpretation. GB participated in data interpretation and manuscript revision. MN participated in the acquisition and interpretation of the data. MAP conceived the study, helped to draft the manuscript and interpret the data. All authors read and approved the final manuscript.
